# Collective Epithelial and Mesenchymal Cell Migration During Gastrulation

**DOI:** 10.2174/138920212800793357

**Published:** 2012-06

**Authors:** Manli Chuai, David Hughes, Cornelis J Weijer

**Affiliations:** Division of Cell and Developmental Biology, College of Life Sciences, University of Dundee, Dundee, DD1 5EH, UK

**Keywords:** Development, chick embryo, chemotaxis, EMT, ingression, FGF signalling.

## Abstract

Gastrulation, the process that puts the three major germlayers, the ectoderm, mesoderm and endoderm in their correct topological position in the developing embryo, is characterised by extensive highly organised collective cell migration of epithelial and mesenchymal cells. We discuss current knowledge and insights in the mechanisms controlling these cell behaviours during gastrulation in the chick embryo. We discuss several ideas that have been proposed to explain the observed large scale vortex movements of epithelial cells in the epiblast during formation of the primitive streak. We review current insights in the control and execution of the epithelial to mesenchymal transition (EMT) underlying the formation of the hypoblast and the ingression of the mesendoderm cells through the streak. We discuss the mechanisms by which the mesendoderm cells move, the nature and dynamics of the signals that guide these movements, as well as the interplay between signalling and movement that result in tissue patterning and morphogenesis. We argue that instructive cell-cell signaling and directed chemotactic movement responses to these signals are instrumental in the execution of all phases of gastrulation.

## INTRODUCTION

It is evident that embryos are self-organising multicellular systems and therefore understanding development will require detailed insights in the control and mechanisms of individual and collective cellular behaviours that result in emergent properties at the level of tissues, organs and the organism. Development is characterised by a well documented sequence of cellular events; fertilisation resulting in formation of the zygote, generally closely followed by a series of rapid divisions to generate many cells. Unequal division of cytoplasmic determinants or asymmetric signalling from extra-embryonic tissues results in an initial diversification of these early cells into a few distinguishable cells types. As cells start to expresses different genetic programmes they acquire the ability to execute different modes of cell-cell signalling and cellular behaviours. Critical cellular behaviours are cell division, programmed cell death, cell shape changes and cell movements. Cell shape changes and movement are especially important during gastrulation where they bring about the large structural rearrangements and cell redistributions that shape the embryo [1]. Besides shaping the embryo these movements create many new cell-cell signalling interactions that are essential to the further diversification of the differential gene expression programmes resulting in a further diversification of cell types and their organisation into tissues and organs. Major research efforts are therefore directed towards defining these diverse cell behaviours, investigation of their integration and control through dynamic cell-cell signalling to obtain novel insights in the emergent phenomena that arise from these interactions. Advances in *in-vivo* imaging make it now possible to follow individual and groups of cells during gastrulation in the context of the embryo and record how and where they move, interact with other cells and their environment and in some cases correlate this with dynamic changes in cell-cell signalling [2, 3]. In this paper we will review our understanding of the role of cell movement during the early stages of gastrulation in the chick embryo, and put this in the context of observations made in embryos of other organisms. 

## BRIEF DESCRIPTION OF IMPORTANT CELL BEHAVIOURS DURING EARLY CHICK DEVELOPMENT

**Fig. (1). Cell flow in epiblast.**
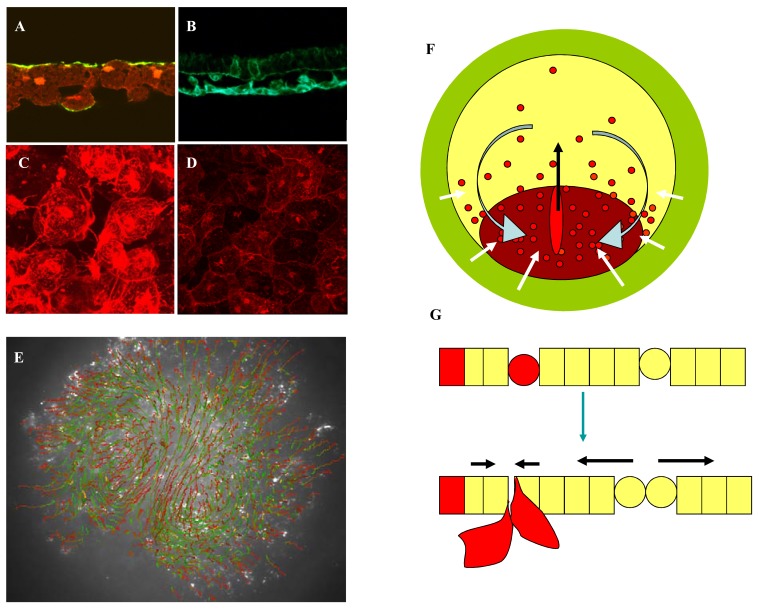
**A**: Section through epiblast of stage EGXII embryo showing the apical localisation of Phospho-ezrin (green) counter staining with Rhodamine Phaloidin to show the actin cytoskeleton). **B**: Section showing two HNK1 positive cells in the epiblast (upper layer) as well as positive hypoblast cells (lower layer). **C**: Hypoblast cells in the anterior of the embryo before fusing to form an epithelial sheet (**D**). **E**: Automated track analysis of fluorescent chicken embryo (1.25x magnification). Cell paths are shown as lines that go from green to red, with a total journey time of 10 hours. The primitive streak and dual circular 'quiet regions' are clearly visible. **F**: Diagram of embryo, green Area Opaca, yellow epiblast. Red dots indicating gradient of cells in epiblast that are HNK1 positive, not that there are more positive cells in the posterior of the embryo than in the anterior. Dark red forming secondary epiblast. Grey arrows indicate cell flow patterns as observed in (**E**). **G**: Diagram showing relationship between HNK1 expression, cell division and ingression. When cells do not express HNK1 divide they stay in the epiblast, resulting in expansion of the epiblast (black arrows) when they express HNK1 and divide they ingress to form hypoblast cells leading to a contraction. The gradients in anterior expansion and posterior ingression result in cell flows indicated by the curved grey arrows in (**F**).

Early chick development starts with the formation of a single cell, the zygote situated on the yolk of a chick egg [1]. This cell undergoes a series of very rapid cell divisions until the time the egg is laid around 24 hours later at which time it contains around “fifty” thousand cells. This indicates that ~15 cell divisions have occurred during these 24 hours, suggesting an average cell cycle time of ~1.6 hrs. The cells are likely to rapidly cycle beween mitosis and DNA replication during these early cleavage stages, but this has so far not been investigated in detail. When after egg laying development resumes the cell cycle is much longer (6-8hrs) and it is assumed that zygotic transcription will start. During the early cleavage phases the cells start to organise themselves into a rough epithelial sheet that is several cell layers thick. The outer rim of cells is continuous with the egg yolk and forms that Area Opaca, which will give rise to the extra-embryonic region of the embryo. The cells in the central area, the Area Pellucida organise themselves in an essentially single layered epithelial sheet that already contains highly polarised epithelial cells [4]. The cells on the outer edge attach to the vitelline membrane, and move outward and keep the embryo under tension [5, 6]. The epiblast cells are highly polarised with a well developed apical basal polarity (Fig. **1A**). The cells have microvilli on their apical side and start to synthesise a basement membrane at their basal side. The cells are connected through well developed tight and adherens junctions. The hypoblast, a transient structure in development, is thought to derive from epiblast through an ingression of individual cells [7]. The hypoblast appears to have an important signalling function during early development, since it has been shown that the hypoblast secretes inhibitors of Wnt and Nodal signalling and through these may play a role in positioning the streak [8]. 

After egg laying, the first large scale movements that take place are coordinated movements in the epiblast and the hypoblast. Cells move from posterior positions in the epiblast to anterior along the central midline and are replaced by cells moving in from more lateral positions [9, 10]. Cells ingress in the area where these cell flows meet, they stack on top of each other and at this stage the streak becomes macroscopically visible. The epiblast portion of the streak extends in anterior direction, while at the same time the deeper regions of the streak start to extend in posterior direction, i.e. streak elongation is bidirectional from the very early stages of development onwards. Once the streak is extended halfway over the epiblast, the cells in the deeper layers of the streak will migrate out to form the endoderm and mesoderm. At this time, the cell movement patterns in the epiblast change and the cells start to flow from lateral positions towards the extending streak. Once the streak is extended roughly 70% over the epiblast, the cells in the tip of the streak change their shape and organisation and form a distinct morphological structure known as the Hensen’s Node [1]. The Node starts to move in a posterior direction, a process known as regression and lays down the notochord and floor plate. Regression coincides with a noticeable posterior expansion of the embryo and initiates development of the embryo from head to tail. During regression the mesendoderm continues to ingress and it appears that the timing of this event is controlled by HOX gene expression as discussed in detail by Durston *et al.* and Woltering in this volume [11].

Dynamic fate mapping experiments have mapped out the migration patterns of the cells in the streak [12-14]. The anterior cells form the notochord and head process, while the cells just behind the streak will from the first pairs of somites. The cells that ingress from the middle of the streak will form the medial and lateral plate mesoderm and the cells in the posterior part of the streak will form the extra-embryonic tissues and particularly the blood vessels in the yolk sac [15]. During the ingression events the cells are in close contact, highly dense and migrate in a collective manner while they frequently make and break contacts with their neighbours. All endodermal and mesodermal precursor cell populations follow very stereotypical migration pathways. These are strongly dependent on external cues since cells from just behind the tip of the streak, that will normally form somites, when transplanted in the posterior streak, that is fated to form extra-embryonic mesoderm and blood-islands will follow essentially trajectories that are characteristic for posterior streak cells [12, 16].

Major questions are: What is the nature of the guidance signals that control all these movements, are they chemical or mechanical signals or a combination of both? What are the mechanisms of movement of cells in epithelial sheets and in their mesenchymal state? We will argue that chemotaxis is an important mechanism operating during these very early stages of development.

## MECHANISMS DRIVING EPITHELIAL CELL FLOW PATTERNS IN THE EPIBLAST DURING STREAK FORMATION

Cell motions are observed macroscopically as flows that centre around two quiet zones in the middle of the embryo and which merge at the site of streak formation [9, 17-19] (Fig. **1E**). The purpose of these movements is to transport the mesoderm, which is induced at the interface between the extra-embryonic and the embryonic area, into the central midline of the embryo. These mesoderm cells form a sickle shaped region at the interface between the extra-embryonic and embryonic regions. It is thought that, in the chicken embryo, the mesoderm is induced by a combination of Wnt and TGF-beta signals coming from a Nieuwkoop centre located in the posterior marginal zone [20, 21]. These mesoderm cells are endowed with different cell behaviours, which are likely to be critical for the execution of these cell flows, since treatments that block mesoderm differentiation also appear to block these cell flow patterns [18, 22]. An interesting but open question is when these rotational movements start, before or during mesoderm differentiation. Several mechanisms have been proposed to explain the observed cell flow patterns. They involve essentially one of the three mechanisms: oriented and or localised cell divisions, cell-cell intercalation and chemotaxis or a combination of any of these [10]. 

### Differential and or Oriented Cell Division 

The first mechanism that was proposed to drive streak formation was localised or oriented divisions as has been proposed for zebrafish [23, 24]. It has been reported that cells in the posterior marginal zone of the chick embryo might show slightly increased cell division rates compared to cells elsewhere in the embryo, based on BrdU incorporation, although the observed differences were only very small [25]. Clonal analysis of cells by viral transfection show that daughter cells form elongated patches in the direction of streak formation [24]. However it is unclear whether this drives or is a result of streak formation. Blocking cell division does not inhibit streak elongation and model calculations have suggested that cell division would not be sufficient to drive streak formation. However both experiment and model calculations have show that cell division alone will help and support the cell flows observed during streak formation [18, 26]. 

### Cell-Cell Intercalation

The second mechanism that has been proposed to control the elongation of the streak is based on cell-cell intercalation of cells in the epiblast [27]. There is some evidence from direct observation of limited cell-cell intercalation during streak formation [19]. It remains to be established whether the intercalation observed is sufficient to explain the observed cell flows, or whether it is a result of these movements. This will have to await detailed quantitative investigation of cell behaviours in the epiblast. The Planar Cell Polarity (PCP) pathway has been shown to be involved in epithelial polarity in fly wings and eyes, as well as in the orientation of hair cells in the cochlea in vertebrates [28]. In dropsophila During fly gastrolation, this pathway does not appear to be involved in reorganisation of the epithelial cells in the blastoderm. 

In vertebrates, especially frogs and zebrafish, the PCP pathway has been described to be involved in the polarisation of mesenchymal cells and proposed to play a causal role in in the intercalation of the mesoderm [29, 30]. Knockdown of several PCP components simultaneously, using a morpholino based approach, showed noticeable effects on streak formation, however it remains to be shown that the knockdown effects were specific and that they affected cell-cell intercalation quantitatively [19]. It is conceivable that PCP components interfere with the apical basal polarity of the epithelial cells and that changes in apical-basal polarity underlie some of the observed effects on cell movement by affecting ingression behaviours as discussed in more detail below [31-33].

### Chemotaxis 

Thirdly it has been proposed that the cells in the epiblast could move in response to chemotactic agents within the plane of the epiblast sheet [10, 17]. Detailed model calculations, using different cell based model formulations, have shown that this is a theoretical possibility. The cells in the streak could either be the source of an attractant and or a repellent, or more effective, a combination of both [34, 35]. A chemotactic mechanism could drive movement of the cells in the streak relative to the surrounding cells. The critical test for this hypothesis will be the unequivocal identification of these in-vivo attractants and repellents, but although there are good candidates (FGF’s, VEGF’s) so far they have remained unconfirmed experimentally. This would require in-vivo knocking down of attractants and or repellents, while observing changes in cell movement and streak formation. Experiments so far have failed to show specific effects due to the fact that these factors are required for differentiation. Knockdown results in loss of mesoderm differentiation as well as movement.

### Cell Shape Change and Ingression

There are other possible mechanisms underlying the cell flows that result in streak formation. One possibility is that the cell movements are driven by differential cell shape changes and or ingression of selected cells in the epiblast. If epithelial cells in the epiblast change their height along the apical basal axis they will change their surface area. Coordinated shape changes can result in local expansion or contraction resulting in cell flows at the scale of the tissue, as has been shown in zebrafish and Drosophila [36-38]. 

In the chick embryo, ingression may also contribute to the observed cell flows There is considerable evidence that the hypoblast forms by differential ingression of a subpopulation of cells in the epiblast [39, 40]. Hypoblast formation starts at the posterior end of the embryo and spreads in an anterior direction as does streak formation, a region of extensive cell ingression [41]. This implies that there is an uneven ingression of cells over the epiblast, with more ingression in the posterior epiblast than anterior and this can contribute significantly to the observed cell flows (Fig. **1F**). The cells that ingress are most likely those that are characterised by expression of the HNK1 antigen. The HNK1 antibody recognises scattered cells in the epiblast and all hypoblast cells (Fig. **1B**). Furthermore HNK1 positive epiblast and hypoblast cells show invasive behavior on extracellular matrix gels and secrete different metalloproteases [42]. Finally, complement mediated deletion of HNK1 positive cells has been shown to inhibit streak formation [43]. Cells that ingress often pull their neighbouring cells towards them, resulting in the formation of transient rosette like structures. These rosette structures are coupled to ingression or cell division [10, 44]. We therefore propose that HNK1 positive epiblast cells ingress when they divide, while epiblast cells that are not HNK1 positive stay in the epiblast when they divide (Fig. **1G**). Since division is random, but there are more HNK1 positive cells in the posterior hypoblast this will generate an effective tissue source in the anterior epiblast and a tissue sink in the posterior, possibly resulting in observed cell flows (Fig. **1F**). Nodal signalling has been implicated in streak formation through its effects on mesoderm differentiation [45]. More recently it has been demonstrated that inhibition of Nodal signalling results in delayed vortex movements and is associated with a reduction in rosette formation, providing further support for a functional link between ingression and streak formation [22, 45]. 

### The Role of the Extracellular Matrix and Movement of the Hypoblast 

It has been shown that the hypoblast can affect the formation of the streak since rotation of the hypoblast results in rotation or bending of the streak [46]. This has been interpreted to imply that the cells in the hypoblast signal to cells in the epiblast and affect their behaviour, for example through modulation of Nodal signalling [8]. It has been shown that, during the early stages of development, the extracellular matrix moves at roughly the same speed as the overlaying epiblast cells [47]. It therefore appears likely that the epiblast cells cannot get any traction from this matrix for their movements. This either implies that the epiblast cells are not moving actively, they are carried by the extracellular matrix, or the epiblast cells have to get traction from their neighbours for all the observed movements [48]. Traction forces would have to be transmitted through these cells to the Area Opaca and also the cells that are in contact with the vitelline membrane and the egg yolk which keep the embryo under tension [5, 6]. Model calculations have shown it to be possible for epiblast cells to get traction from other epiblast cells [35]. If the movement of the hypoblast cells is mechanically coupled to movements in the epiblast, for example through coupling through the ECM, the question becomes what is it that drives the movements in the hypoblast? Hypoblast cell movement could be driven by two Mechanisms: flattening of the initially round loosely connected hypoblast cells during epithelial sheet formation, which results in an increase in their surface area or active cell movement (Fig. **1C**,**D**). It has been shown that during mouse gastrulation the anterior visceral endoderm (AVE), the homologue of the chick hypoblast, migrates in a directional manner and that the protrusive behaviour of the AVE cells requires the activation of the small G protein Rac which is most likely required for the control of actin polymerisation in the leading edges of the cells [49-52]. This active migration results in expansion of the epiblast in mouse and in the chick could be driving the anterior movement of the streak. 

## EMT AND THE MECHANISM OF CELL INGRESSION THROUGH THE PRIMITIVE STREAK

### Control of the Site of Ingression 

As the streak starts to form, the cells in the streak already start to undergo an EMT, a process that continues during further development [53, 54]. Cells that ingress in the streak show a strong contraction of their apical sides and elongate basally. It would appear most likely that the apical contraction is a MyosinII driven process as this has been shown to be the case in other organisms especially Drosophila [55-57]. In Drosophila the apical constriction of invaginating cells in the ventral furrow is spatially controlled by a dedicated signalling system. Furrow formation is controlled by a secreted peptide ligand, Folded gastrulation (Fog), that interacts with a G protein coupled serpentine receptor that in turn results in the activation of a G protein (Concertina). This then results directly or indirectly in the activation of a RhoGef (RhoGef2) which through Rho and Rho kinase results in the activation of MyosinII contraction [58]. It seems evident that in vertebrates there also has to exist a mechanism that instructs cells where to ingress. In vertebrates all the components for a system homologous to the Drosophila signalling cascade such as homologues of the G protein coupled serpentine receptor, the G protein heterotrimeric alpha subunit, a RhoGef and all the components required to signal to MyosinII are present during gastrulation, however a clear homologue of Fog remains unknown. 

### The Mechanisms of Ingression

**Fig. (2). Cell ingression in the primitive streak.**
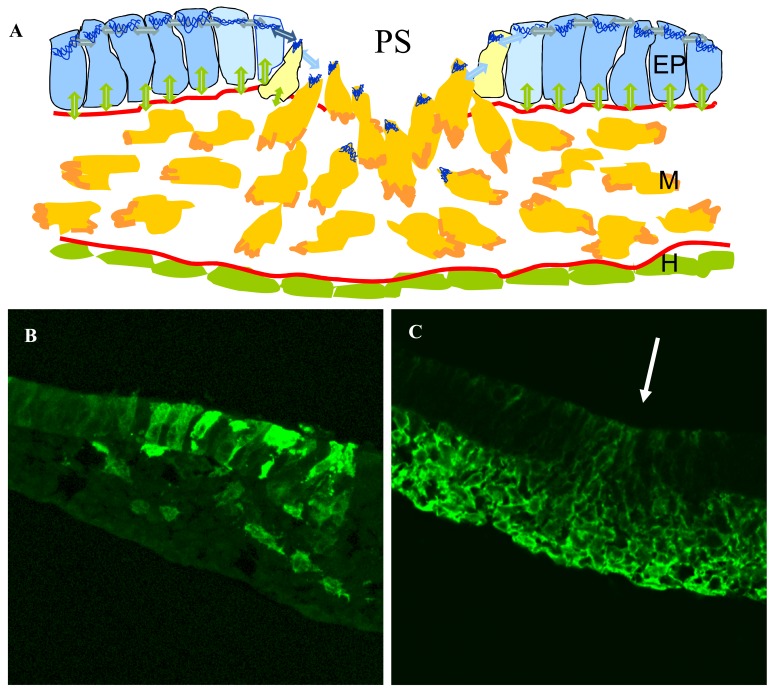
**A**: Diagram showing changes occurring during ingression in the streak. A signal secreted by the mesoderm cells (light-blue and yellow) results in apical contraction of these cells by myosin (apical blue line). Simultaneously the cells reduce the strength of E-Cadherin medicated interactions (blue double arrows between cells), break down the basal lamina (redline) and down regulate integrin mediated signalling to the matrix ( (green double arrows), polarise by synthesizing actin at their leading edges (orange) and migrate in the internal space of the embryo in response to repulsive ad attractive guidance factors. **B**: Diagram showing changes in cells shape of random GFP labelled cells from being columnar in the epiblast to being polarised in the direction of migration of mesoderm cells. **C**: Section of streak region (white arrow) showing expression of N cadherin. Note that cells in the streak start to express N-Cadherin as do all the mesendoderm cells.

The detailed cellular mechanisms that drive ingression in the chick are not yet fully resolved. It seems evident that ingression requires myosin mediated apical contraction and disassembly of the basal membrane. The latter appears to be regulated through a Rho dependent pathway, most likely through microtubule dependent changes in anchorage and possibly secretion of dedicated metallo-proteases [53]. How the cells physically move down is, however, still largely unresolved. It could be that the cells polarise and migrate down actively, using surrounding cells as the substrate to crawl on. In this case the basal side of the cells must act as the leading edge of the cell and the former apical end of the cells becomes the back (Fig. **2A**). There are as yet no clear polarity indicators, however if ingression involves an active migration process it would be expected that cells would show increased actin polymerisation at their leading edge and coordinated myosin contraction in the back of the cell as is typical for individual migrating cells [59, 60]. Experiments performed with specific inhibitors indicated that MyosinII action is required for successful extension of the body axis during streak formation [17] as is well known from frogs, fish and Drosophila [56, 57, 61] suggesting that MyosinII function is critical for effective cell movement. Directional movement would depend on specific guidance factors as discussed below (Fig. **2B**). Alternatively it could be imagined that cells modulate cell-cell adhesion to drive differential movement. A basal to apical flow of N-Cadherin molecules has been shown to exist in cultures of epithelial cells, most likely driven by coupling to the cytoskeleton [62]. In chick, embryos ingressing mesoderm cells express N-Cadherin (Fig. **2C**). If the speed of flow of N cadherin was differentially upregulated in an ingressing cell compared to its neighbours then this flow of adhesion molecules would push the cell down relative to its neighbours and thus result in effective ingression. In-vitro systems show that RAS transformed cells can either exit the epithelial cell layer apically or basally. This appears to be controlled by the expression level of E-Cadherin in combination with activation of signalling cascades to the actin cytoskeleton through small G proteins such as Ras and CDC42. When surrounding cells express reduced amounts of E-cadherin cells are more likely to egress basally [63]. The so called Cadherin switch, which involves a downregulation of E-cadherin and simultaneous upregulation of N-cadherin has been proposed to be key driver for EMT in many biological systems [64, 65]. In mice the FGF-Snail pathway is thought to control the E- to N-Cadherin switch through Snail mediated transcriptional repression of E cadherin [65-67]. Whether this is important in chick EMT remains to be established since few direct effects of inhibition of FGF signalling on E cadherin expression have been observed so far [68]. In mouse it has recently been shown that signalling though a Ste20 kinase pathway is required for cells to ingress through the streak and that this may act in parallel with the FGF-Snail mediated pathway. We have manipulated the PI3 kinase signalling pathway, through the use of inhibitors and expression of dominant negative PTEN constructs. Expression of a membrane targeted PTEN can completely inhibit EMT. One possible interpretation is that this is due to the constitutive dephosphorylation of a membrane bound protein substrate (beta catenin), which is necessary for effective coupling of adhesion sites to the cytoskeleton [69, 70].

## THE MECHANISM OF MOVEMENT OF MESENCHYMAL CELLS AFTER THEIR INGRESSION THROUGH THE STREAK

**Fig. (3). Migration of GFP labelled mesendoderm cells.**
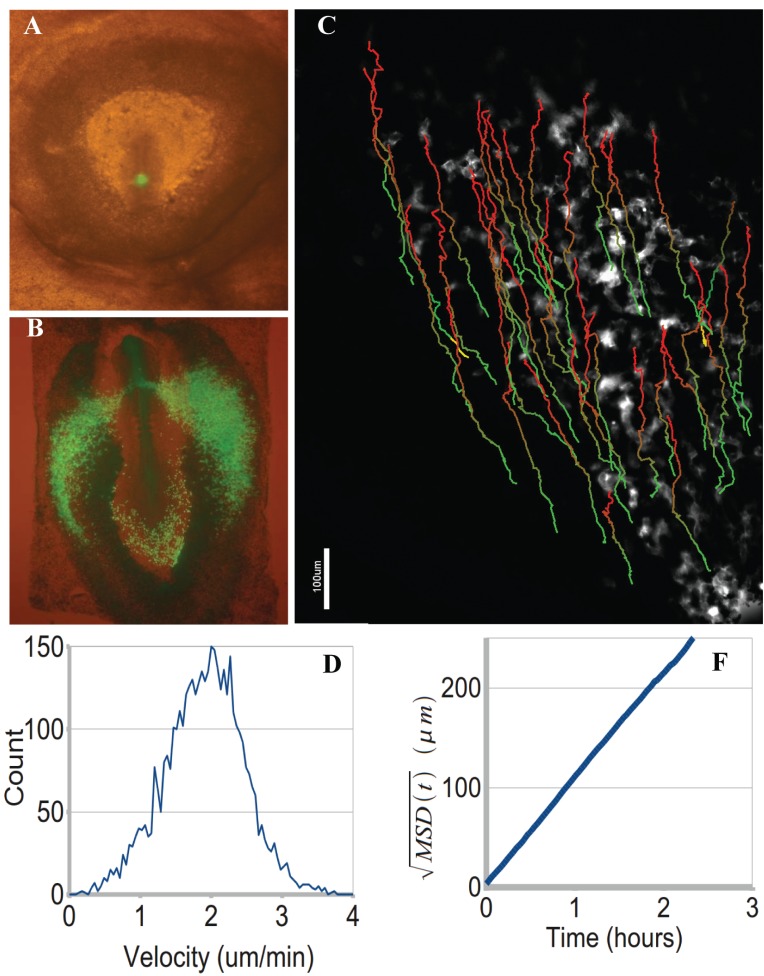
**A**: Image of a HH3 stage embryo with a small graft of GFP expressing streak cells from a transgenic embryo. **B**: photograph of the same embryo after 24 hrs of development (HH7) showing that the GFP cells have divided extensively and migrated to form precursors of the blood islands and blood vessels as well as some cells in the posterior of the embryo that have form endoderm. **C**: Image of tracks of grafted GFP labelled mesoderm cells migrating out of the primitive streak (10 x magnification). Automated track analysis displayed as linear paths coloured from green to red, showing a total journey time of 7 hours. **D**: Velocity distribution of cells imaged in **C**. **D**: Square root of meansquare displacement of cells tracked in (**C**) showing a linear increase of distance with time showing highly directed migration.

Cells in the streak ingress and after their ingression move away to form the different endodermal and mesodermal derivatives (Fig. **3A**, **B**). This involves high stereotypical movements over large distances. Important questions are: How do cells know where to go? Do they have internal programmes or do they respond to external signals? Do the migrating cells signal to surrounding cells as they pass by, i.e. is there a feedback of the migrating cells on signalling by other cells and what are the mechanisms of migration? Below we will discuss some of the experimental results that address some these questions and discuss their implications.

Heterotypic grafting experiments where cells from the anterior streak were placed in the posterior streak and vice versa have shown that cells move as dictated by their position [12]. This suggests that the cells move mainly in response to cues or signals present in the extracellular environment. The second major question is whether the cells move in response to chemical or mechanical cues or possibly to a combination of both. This is a more difficult question to answer. Cells that migrate out from the primitive streak initially colonise a relatively empty region between the epiblast and the forming endoderm. The cells migrate at high density and are continually in close contact. They appear to migrate in a very directional manner with an average speed of 2um/minute (Fig. **3C**, **D**, **E**). 

### Guidance by Chemotaxis

**Fig. (4). Signal and cell migration patterns during gastrulation.**
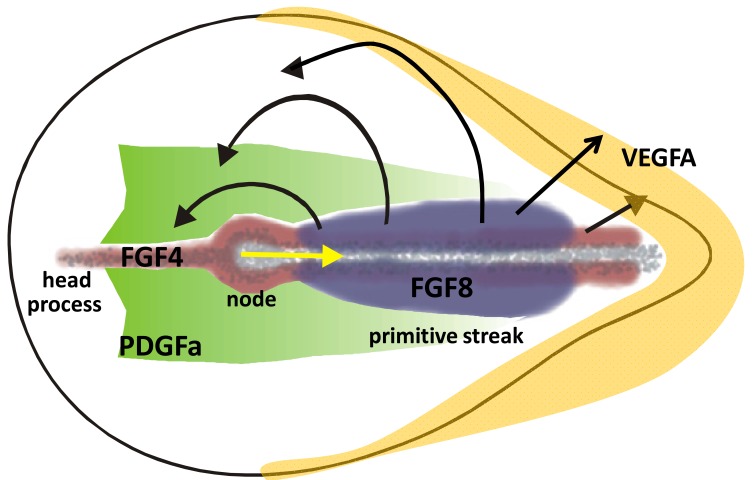
Black arrows indicate movement trajectories of mesendoderm cells migrating out of the streak at various anterior to posterior positions in. Cells move out of the streak under the influences of a repulsive FGF8 signal, anterior mesendoderm cells move back in response to FGF4 produced by the forming head process and notochord. Cells in the posterior streak move in the extra-embryonic area to form blood islands response to VEGF. PDGF signalling in the epiblast controls N-Cadherin expression of migrating mesoderm cells and lays out a migration domain for mesoderm cells.

Once the cells start to move out of the streak they have to know where to go to make any meaningful structure. It has been shown that mesoderm cells are very sensitive to FGF4, a factor that is secreted by cells in the forming notochord and head process during the early stages of gastrulation (up to the formation of 3-5 somites). Mesoderm cells from the anterior part of the streak show a striking chemotactic response to FGF4 and this response is inhibited when these cells express a dominant negative FGFR1 receptor. It was also shown thatmesoderm cells are repelled by FGF8, which is abundantly expressed in the streak [12]. Cells ingression through the streak move away from a localised FGF8 source, which is in agreement with observations in mice which show that FGF8 knockout embryos show a defective migration of mesodermal cells [71]. Therefore the migration of mesoderm cells would be controlled by a balance between chemo-repulsion and chemo-attraction. It would appear that cells situated in the streak just behind the node migrate out but soon will come under the influence of the FGF4 secreting forming head-process and notochord and migrate back (Fig. **4**). Cells that migrate out from the middle streak keep migrating for longer before they can sense the FGF4 signal secreted by the regressing node and forming notochord. They start to move back but never return completely toward the central midline, since these positions are already taken by the cells from more anterior positions in the streak. These cells however do not cross the boundary of the embryo and do not leave the embryonic area. In frogs it has been be proposed that PDGF signalling is an important mechanism to control the anterior migration of the leading edge mesoderm cells [72, 73]. Arguments have been presented to show that gradients in PDGF are required. We have shown in the chick embryo that PDGF signalling is required for cell movement of mesoderm cells [74]. However we don’t think that PDGF gives *in vivo* information to the cells where to go, but through its effect on cadherin expression defines a mechanism that is responsible for laying out an area where cells can move effectively. Outside this area the expression of N-cadherin is unstable and the mesoderm cells loose expression of N-Cadherin on their cell surface and fail to get enough traction to move. Therefore we envisage PDGF signalling to lay out a potential migration territory, while the fine tuning of migration is achieved by other factors. More recently it has become evident that FGF signalling plays a key instructive role also in the migration of mesoderm cells in more primitive organisms such as Drosophila and Ciona and it therefore appears to be an evolutionary highly conserved mechanism [75-78]. This mechanism of repulsive and attractive responses to FGF signals results in a translation of the original anterior-posterior organisation of cells in the epiblast of the streak into a medial to lateral organisation of cells in the mesoderm. 

Cells that leave the most posterior streak are attracted by factors such as VEGF. This appears to be synthesised in the deeper layers of the extra-embryonic area and acts as an attractant for these cells. In the extra-embryonic area the cells aggregate to form blood islands which in turn fuse to form blood vessels [16, 79]. Blocking the VEGFR2 receptor blocks the migration of these cells away from the streak. There is the possibility that the cells at the leading edge of a migrating cohort of cells sense a signal and that they then secrete another signalling molecule that allows other cells to follow them, i.e. a relay mechanism as has been shown to be the case in cancer cell metastasis [80].

It seems likely that many other factors will be involved in the migrate of cells when they leave the primitive streak. It has been suggested that cells migration away from Wnt5B signals and that these effects may be mediated though the tyrosine kinase receptor RYK [81, 82]. Through studies, mostly in other organisms, it is becoming increasingly clear that signalling though small peptides may also provide important information for the migration of mesoderm cells. In zebrafish it has been shown that signalling through Gα12/13 is required for mesoderm cells to migrate towards the midline [83]. While in Xenopus it has become clear that cytokines especially SDF1 signalling though the G protein coupled serpentine receptor CXCR4 and CXCR7 is important for mesendoderm cells to migrate towards the midline [84, 85]. It seems likely that these molecules will also play a role in gastrulation in amniotes such as the chick.

### Guidance by Chemokinesis 

Cell movements have also been analysed in considerable detail in the later stages of gastrulation, when segmentation is well underway. These investigations monitored both the movements of the cells in the presomitic mesoderm and the movements of the extracellular matrix as detected by antibody labeling of the matrix. It was found that the matrix moves in similar direction as the cells and that the cells migrate only a limited amount relative to the surrounding extracellular matrix [86]. One interpretation of these experiments is that a substantial part of the mesoderm cell movement observed at these stages of gastrulation is passive, resulting from deformations of the ECM, possibly caused by shape changes elsewhere in the embryo. By considering the movement of the cells relative to the average matrix movement only a small active randomly orientated movement component is left. This has been taken to signify that the active movement can effectively be described as a random walk. Furthermore it was observed that the cells in the posterior presomitic mesoderm at the high point of the FGF gradient, that controls segmentation, move at a significantly higher speed than the more anterior cells in the lower concentrations of FGF8. This could imply a chemo-kinetic mechanism, where the intensity of cell motility is controlled by FGF8, but where the cells do not read the concentration gradient. Chemokinesis will result in cells stumbling down the FGF8 gradient in a random manner, but it is relatively inefficient at longer space scales and it is more potentially difficult to understand how this can control the formation of specific structures.

To distinguish between chemotaxis and chemokinesis it is necessary to know how much of the cell movement is active and how much is passive. In chemotaxis all active movement is directional, while in chemokinesis active movement follows a random walk. So far it has proven difficult to asses how much cells move actively and how much passively. It all will depend on determining whether cells get traction from each other or the matrix as discussed above. If the cells move actively then it would mean that the extracellular matrix deposited serves a different purpose than acting as a material to get traction from, it possibly serves a role in signalling. 

Finally the observed posterior-anterior gradient in active cell movement gradient was postulated to drive the elongation of the embryo. It however remains to be established whether this mechanism can deliver the forces required to deform the embryo.

## CONCLUSION AND OUTLOOK

One of the main aims of understanding morphogenesis in an embryo as complex as the chick embryo is to understand the interactions between cell-cell signalling, differentiation and cell behaviours such as cell-division, -death, -shape changes and movement. In the case of the chick embryo this involves understanding cellular behaviours during the phase of development where cell number of cells increases from 50.000 to 300.000 cells organised in a few distinct tissues and possibly up to a dozen cell types. Although considerable progress has been made in monitoring the in-vivo migration of tens to thousands of cells, which will enable us to get to grips with their behaviours, it has been very difficult so far to analyse the in-vivo signalling dynamics that control these cell behaviours. This is why at present a wide range of different hypotheses still persist about how the basic machinery of gastrulation works. A further challenge will be to get more mechanistic insight in the cellular processes driving the observed cell movements, especially in the question of how much of the movement is active or passive. It is however rapidly becoming clear that cells can move by a well understood cellular mechanism of chemotaxis and that this may underlie many of the phenomena observed during gastrulation. It is clear that all members of the split kinase domain family of receptors that code for FGF, PDGF and VEGF signalling are potent mediators of chemotactic responses as well as members of the family of Wnt receptors and co-receptors. Signalling through small molecules (chemokines, lipids) through G protein coupled receptor may turn out to be important in the precise control of migration of specific cell types adding to the complexity. The ultimate goal will be to understand the dynamic interactions between signalling and movement that result in gastrulation.
